# Functional characterization of two KCND3 variants associated with SCA 19/22 ataxia in Latin American families

**DOI:** 10.1186/s40659-025-00589-3

**Published:** 2025-03-26

**Authors:** Felipe Arancibia, Fernanda Martin, Jenny Ruiz-Fuentes, Erbio Diaz, Tamara Hermosilla, Wendy Gonzalez, Felipe Simon, Diana Avila-Jaque, Mariana Luna-Álvarez, David José Dávila Ortiz de Montellano, Marcelo Miranda, M. Leonor Bustamante, Diego Varela

**Affiliations:** 1 Programa de Fisiología y Biofísica, Instituto de Ciencias Biomédicas, Facultad de Medicina, Universidad de Chile, 8380453 Santiago, Chile; 2 Millennium Nucleus of Ion Channels-Associated Diseases (MiNICAD), Universidad de Chile, Santiago, Chile; 3 Center for Bioinformatics, Simulations and Modelling (CBSM), University of Talca, 3460000 Talca, Chile; 4 Laboratory of Integrative Physiopathology, Faculty of Life Sciences, Universidad Andres Bello, Santiago, Chile; 5 Hospital San Juan de Dios, Santiago, Chile; 6 Unidad Asesoramiento Genético Oncológico, Fundación Arturo López Pérez, Santiago, Chile; 7 Instituto Nacional de Neurología y Neurocirugía Manuel Velasco Suárez, Mexico City, Mexico; 8 Fundación Diagnosis, Santiago, Chile; 9 Clínica MEDS, Santiago, Chile; 10 Programa de Genética, Instituto de Ciencias Biomédicas, Facultad de Medicina, Universidad de Chile, 8380453 Santiago, Chile; 11 Millennium Institute On Immunology and Immunotherapy, Santiago, Chile

**Keywords:** *KCND3*, Kv4.3, Spinocerebellar ataxia SCA19/22, Congenital ataxia, Functional characterization

## Abstract

**Background:**

Spinocerebellar ataxia 19/22 (SCA19/22) represents a rare autosomal dominant genetic disorder resulting in progressive ataxia and cerebellar atrophy. SCA19/22 is caused by variants in the *KCND3* gene, which encodes a voltage-gated potassium channel subunit essential for cerebellar Purkinje cell function. To date, 22 variants have been reported worldwide, with incomplete functional studies.

**Results:**

We present four Chilean and Mexican cases in whom two single-nucleotide variants were identified through whole-exome sequencing of the probands. One variant (G371R) was initially cataloged as pathogenic and the other (S357W) as likely pathogenic according to the American College of Medical Genetics and Genomics criteria. The pathogenicity of the G371R variation was confirmed by *in-silico* mutagenesis. Our molecular models, that include electrostatic potential analysis and algorithms to analyze the pore dimensions (HOLE), indicated that the longer side chain of the arginine narrowed the channel’s selectivity filter, while the positive charge modified its surface electrostatic potential, presumably preventing potassium flux. Functional characterization of the S357W variant was performed in AD293 cells. When overexpressed, K_V_4.3^S357W^ channels alone showed no current. Protein electrophoresis revealed that the total number of K_V_4.3 channels expressed did not differ between the wild-type and mutated phenotypes, suggesting a protein trafficking malfunction. Co-expression of the KChIP2 auxiliary subunit partially rescued the potassium currents when the variant was expressed, albeit with very different biophysical characteristics, including faster inactivation vs. wild-type channels.

**Conclusions:**

This functional characterization of two *KCND3* variants associated with SCA19/22 adds new evidence for the pathogenic role of Kv4.3 loss-of-function mutations and establishes a correlation between functional dominance and clinical severity in SCA19/22.

## Background

Spinocerebellar ataxia 19/22 (SCA19/22) is a rare genetic disorder described for the first time in 2012 [1, 2]. Currently, approximately 100 patients, from European and Asian ancestries has been diagnosticated with this type of ataxia, and recently, our group report the first cases in Latin American individuals [3]. Progressive ataxia with cerebellar atrophy is the typical presentation [4]. It is inherited in an autosomal dominant pattern and is caused by mutations in the *KCND3* gene, located at chromosome 1p13.2, which encodes for the Kv4.3 voltage-dependent potassium channel; currently 22 different variants (most of them missense) have been reported [5]. This channel plays a crucial role in the function of cerebellar Purkinje cells controlling the excitability level of these cells.

Kv4.3 channels underlie somatodendritic A-currents in several types of neurons and the transient outward potassium current (Ito) in cardiac myocytes. This subthreshold K^+^ outward current is activated by membrane depolarization and rapidly inactivates. Kv4.3 channels can affect membrane repolarization and thereby modulate the frequency of slow repetitive firing or backward spread of action potentials, thus preventing neuronal excitability [6, 7]. In the heart, Ito shapes the early phase of repolarization and sets the plateau voltage level of the action potential [8].

Loss-of-function of this channel has been associated with motor coordination impairment, which leads to ataxia and other neurological symptoms [1]. The most commonly reported SCA19/22 phenotype is slowly progressive cerebellar ataxia, which is generally characterized as a pure cerebellar phenotype with predominant gait disturbance. Problems with balance are usually the first symptoms, followed by dysarthria, nystagmus, and later dysphagia [9]. However, a diverse clinical spectrum has been described, including other neurological features such as cognitive impairments and a wide range of movement disorders, including myoclonus, dystonia, and parkinsonism [2].

To date, less than 20 *KCND3* single-nucleotide mutation sites associated with SCA19/22 have been reported worldwide, with no cases reported in Latin America [5]. These missense mutations result in functional consequences, leading to channel function impairment due to changes in channel expression levels [2, 9–12], reduced channel trafficking to the plasma membrane [2, 9–11], or changes in the biophysical properties of the channel [2, 10–14]. Nevertheless, despite accumulating functional data, no clear link has been established between clinical severity and the functional consequences of the variants on Kv4.3 channel activity.

We herein present four SCA19 cases from two families in Chile and Mexico. Whole-exome sequencing analysis revealed novel variants for both families. Co-segregation analysis suggested a de novo variant in the Mexican family resulting in a G371R missense mutation in the Kv4.3 potassium channel. In the Chilean family, the analysis pointed to a nucleotide substitution showing a pattern consistent with autosomal dominant hereditary associated with the S357W variant in *KCND3*. *In-silico* and functional analyses of these variants indicated decreased channel function in the plasma membrane. These findings add new evidence for the pathogenic role of Kv4.3 loss-of-function mutations.

## Methods

### Patients

All clinical assessments were performed under the supervision of a neurologist with experience in movement disorders. Genetic testing was carried out after counseling by the attending physician and genetic counselor as appropriate. Samples were submitted to clinically accredited laboratories abroad, and quality control and bioinformatic analyses were completed according to standard procedures in each laboratory. All patients subsequently provided signed informed consent for their records to be used in research protocols approved by the local IRB (Chile and Mexico), including their consent for publication. Pre- and post-testing genetic counseling was provided to ensure that each participant understood the potential results and the limitations of the testing.

### Constructs

cDNA encoding the Kv4.3 potassium channel was kindly provided by Dr. J. M. Nerbonne. The HA epitope (YPYDVPDYA) was inserted by conventional PCR after glycine 212, corresponding to the S5 end. The Kv4.3 p.S357W variant was created by using the two-step PCR protocol. The primers list used is detailed in Table 1. In-frame insertion of the HA-tag and correct amino acid change was confirmed by DNA sequencing; no differences in biophysical properties were found as compared to wild-type channels (not shown). The KChIP2 clone was kindly provided by Dr. S. A. N. Goldstein.

**Table 1:** List of primers used in the present study

Name	Sequence (5′ to 3′)
HA_Tag.FOR	tacccatacgatgttccagattacgctccgtgcggggagcgctactc
HA_Tag.REV	agcgtaatctggaacatcgtatgggtacagctccttgctgcccggga
KCND3.REV	agccgaagatcttccctgcaatcg
KCND3.FOR	tgcaggaattcatggcggccggag
KCND3.REV2	atccctcgagttacaaggcggaga
S357W.FOR	aagcatccctgcctggttttggtacacc
S357W.REV	ggtgtaccaaaaccaggcagggatgctt

### Kv4.3 system visualization

The structure of Kv4.3 (PDB: 7W3Y) is publicly available and was obtained from the RCSB Protein Data Bank. Molecular illustrations were generated using VMD [15] and PyMOL [16] software.

### *In-silico* mutagenesis

The wild-type residues (S357 and G371) were mutated computationally using PyMOL [16] The HOLE algorithm was used [17].

### Electrostatic potential analysis

Electrostatic potential analysis was performed with the Adaptive Poisson–Boltzmann Solver (APBS) [18] on the APBS-PDB2PQR software suite web server (APBS-PDB2PQR Software Suite; available online: https://server.poissonboltzmann.org/ (accessed on 21 October 2023). The PDB2PQR tool was used to prepare the structures for subsequent electrostatic analysis, adding atomic charge and radius parameters to PDB data and estimating titration states and protonation of the residues. APBS solves the equations of continuum electrostatics for biomolecular assemblies, calculating electrostatic and solvation properties, through the implicit Poisson–Boltzmann (PB) solvent model, which provides a global solution suited to visualization and structural analyses.

Spatial distribution of the electrostatic potential was calculated at physiological ionic strength (I) = 150 mM, assuming + 1/ − 1 charges for the counter-ions. Partial charges and Van der Waals radii were assigned via PDB2PQR according to the AMBER force field [19], choosing an interior dielectric of εp = 2 for proteins and εs = 78.54 for the solvent. The temperature was set at T = 310.15 K, and the solvent probe radius was set was set at r = 1.4 Å. Isopotential contours were plotted at ± 5 kBT/ec and viewed using PyMOL [16]. The preferred unit of electrostatic potential is kBTec − 1 or RTec − 1. If T≈310 K, then kBTec − 1 = RTec − 1 ≈ 0.0256JC − 1 = 26.7 mV.

### AD-293 culture and transfection

Tissue culture of AD-293 cells (Agilent) was performed as recommended by the distributor. Briefly, AD-293 cells were grown in Gibco Dulbecco’s modified Eagle’s medium (DMEM) (Thermo Scientific,Waltham, MA, USA) with 5% fetal bovine serum (Thermo Scientific, Waltham, MA, USA), 100 units/mL penicillin–streptomycin, and maintained at 37° C in a humidified incubator with 95% air and 5% CO_2_. Cells were split before reaching 90% confluence, only passages under 25 were used. Transfection solutions for individual culture dishes (35 mm diameter) contained a mixture of cDNA expression vectors (500 ng for each potassium channel subunits and 50 ng of EGFP as reporter) and were transfected into cells by lipofectamine 2000 according to manufacturer instructions. Experiments were conducted at room temperature 16–40 h after transfection.

### Western blot

Western blot Cells were lysed in cold hypotonic solution (50 mM TRIS, 1% IGEPAL, 150 mM NaCl, 1 mM EDTA, pH 7.4, and protease inhibitors) for 1 h under constant agitation. Cell lysates were centrifuged at 13,000 rpm for 10 min [20]. Proteins were separated by electrophoresis with 8% SDS-PAGE gels for 3 h at 100 V, and subsequently transferred to nitrocellulose membrane. The nitrocellulose membrane was blocked with 5% skim milk in PBS 1X. Kv4.3 was identified using a monoclonal antibody vs HA-Tag (SC-7392) (Santa Cruz) at a concentration of 1:200 and tubulin with a monoclonal antibody (DM1A) (Novus Biological) at 1:1000. A 1:2500 anti-mouse IgG, HRP-linked antibody (Cell Signaling) was used as a secondary antibody. Proteins were visualized by ECL. (Thermo Scientific).

### Electrophysiology and data analysis

K^+^ currents were recorded by conventional whole-cell patch clamp [21, 22]. Borosilicate glass pipettes were pulled to 2–4 MΩ resistance and filled with internal solution containing (mM): 100 KCl, 5 MgCl_2_, 10 EGTA, 10 HEPES, 2.5 Na_2_ATP (pH 7.4 adjusted with KOH). Bath solution contained (mM): 140 NaCl, 5 KCl, 1.2 MgCl_2_, 2 CaCl_2_, 10 HEPES, 5 glucose (pH 7.4 adjusted with NaOH). Data were acquired at room temperature using an Axopatch 200B amplifier and pClamp 10 software (Axon Instruments), low pass-filtered at 5 kHz, and digitized at 10 kHz. Data analysis, current fitting, and offline leak subtraction were performed in Clampfit 10 (Axon Instruments) and SigmaPlot 11 (Jandel Scientific).

Conductance-voltage graphs were constructed after recording the potassium current by applying voltage pulses from − 80 to 80 mV from a holding potential of − 80 mV in 10 mV increments. The conductance for each voltage was calculated as the maximum current amplitude divided by the reversal potential extrapolated from a linear regression of the current. Each dataset was normalized to the maximum value obtained in each experiment.

Normalized conductance-voltage (GV) plots were fitted using a modified Boltzmann equation:

$$\frac{G}{{G}_{max}}=\frac{\left(V-{E}_{rev}\right)}{1+{e}^{-\left(V-{V}_{a}\right)/k}}$$

where E_rev_ is the reversal potential, G_max_ is the maximum slope conductance, *k* is the slope factor, and V_a_ the half-activation potential. Mean current density plots were generated by measuring the maximum current amplitudes as a function of voltage and normalized to cell capacity determined by a short hyperpolarizing pulse before the main pulse. Data from at least four different transfections were then averaged.

### Statistics

Data are presented as mean ± SEM (n). Statistical analysis was performed with SigmaPlot 11 (Jandel Scientific) using unpaired Student’s t-tests, and results were considered significant at p < 0.05. One-way ANOVA tests were performed for samples exposed to multiple treatments, and results were considered significant at p < 0.05.

## Results

### Clinical description and initial assessment

The clinical features of all reported carriers of the variants have been described elsewhere [23]. A summary of clinical findings is presented in Table 2.

**Table 2:** Clinical overview of individuals carrying the KCND3 variants analyzed in this study

Cases	Proband (Chile 1)	Father (Chile 1)	Brother (Chile 1)	Proband (Mexico)
Variant	*KCDN3*: c.1070C>G	*KCDN3*: c.1070C>G	*KCDN3*: c.1070C>G	*KCND3:* c.1111G>A
Gender	Male	Male	Male	Male
Year of birth	1973	1936	1971	1991
Age at examination	48 yrs	85 yrs	50 yrs	31 yrs
Initial presentation	Ataxia	Ataxia	Ataxia	Developmental delay
Ataxia age of onset	19 yrs	35 yrs	29 yrs	25 yrs
SARA score	9,5	23	17	14
Movement disorder besides ataxia	Head tremor	No	No	No
Epilepsy	No	No	No	No
Cognitive impairment	No	Yes	No	Yes. Intellectual disability
MOCA score	24	20	27	N/A
Neuroimaging	Cerebellar atrophy of vermis and hemispheres	Diffuse cerebellar atrophy brain involution bihemispheric microangiopathic changes	Cerebellar atrophy Right vascular infarct sequelae Diffuse brain involution	Cerebellar atrophy
Electroencephalogram	Normal	Normal	Normal	Normal
Electromyography	No signs of polyneuropathy	Large fiber distal sensory polyneuropathy	No signs of polyneuropathy	No signs of polyneuropathy
Genetic testing	Gene panel; WES	Gene sequencing	Gene sequencing	Karyotype; aCGH; WES

**Fig. 1:**
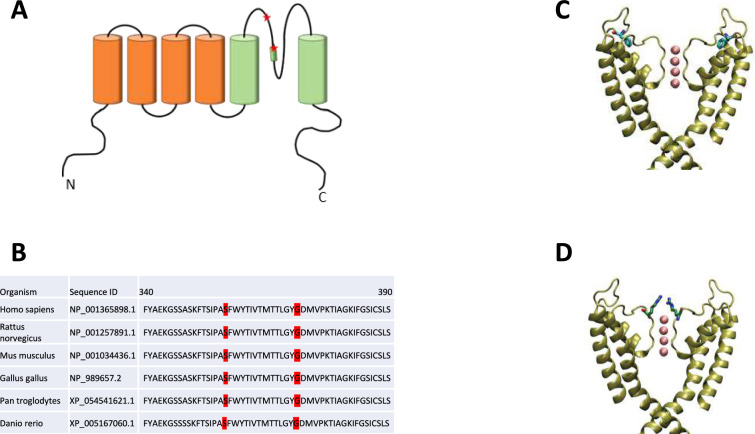
Protein structure modeling of human p.S357W and p.G371R variants: (**A**) Schematic diagram of the membrane topology of a Kv4.3 subunit containing six transmembrane segments (S1–S6). The localizations of the two variants studied in this work are highlighted. **B** Amino acid sequence alignment of various Kv4.3-relvent homologs and orthologs showing that the residues studied in this work are highly conserved. **C** and **D** Structural models of Kv4.3 variants based on the Kv4.3 structure available in the RCSB Protein Data Bank (PDB: 7W3Y). For clarity, the N-terminal of Kv4.3 channel was removed up to residue 318, and chains B and D are not shown. S357W (**C**) and G371R variants (**D**) shown in licorice

Proband 1 was a Chilean male with slowly progressive ataxia beginning at 19 years of age. His father and one brother also showed ataxic symptoms. After a negative screening for the most common repeat expansion variants known to cause SCA, whole-exome sequencing (WES) was performed for the proband. Results revealed a heterozygous variation in exon 2 of the *KCND3* gene (NM_004980.4:c.1070C > G), which resulted in the substitution of serine by tryptophan at position 357 in the P-loop of the channel (Fig. 1A). The presence of this variant was confirmed in both affected relatives (also in a heterozygous state) through gene sequencing.

Proband 2 was a 30-year-old male, the first son of non-consanguineous healthy parents. He presented with developmental delay in early life and developed an ataxic gait at the age of 25. Brain MRI showed generalized cerebellar atrophy. Karyotype and subsequent microarray-based comparative genomic hybridization (aCGH) showed normal results. WES revealed a heterozygous variation in exon 2 of the *KCND3* gene (NM_004980.4:c.1111G > A), which resulted in the substitution of glycine by arginine at position 371, which corresponding to the last glycine of the signature sequence of voltage-gated potassium channels (GYG) [24], located at the outer pore region as depicted in Fig. 1A. The variant was classified as pathogenic according to the American College of Medical Genetics and Genomics (ACMG) criteria [25] because it represents a missense variant in a gene with a low rate of benign missense variation wherein missense variants are a common mechanism of disease. The variant was absent from genetic variation databases, and *in-silico* sequence alignment predicted that the change was deleterious (PP2 criteria); moreover, it was previously reported as likely pathogenic in ClinVar [26]. Neither of the parents carried the variant, suggesting a de novo origin of the mutation.

Protein sequence alignment of multiple species reveals that both amino acids are highly conserved, as shown in Fig. 1B. Additionally, protein modeling, based on the cryo-EM structure of human Kv4.3 [27], indicates that substituting the polar serine by the nonpolar tryptophan causes an increase in volume and hydrophobicity at the beginning of the P-loop (Fig. 1C). Conversely, a mutation at residue 371 replaces the amino acid with the smallest side chain with arginine, a charged amino acid which comprises a 3-carbon aliphatic straight side chain in the signature sequence, thereby reducing the pore size (Fig. 1D).

### *In-silico* results

The TLGYG sequence is responsible for the high selectivity of Kv4.x channels (Fig. 1B and [28]). This sequence is highly conserved in all K^+^ channels and consists of five consecutive coordination sites for K^+^ at the channel pore that are chemically equivalent (S0-S4). These coordination sites orient the partial negative charge of the carbonyl oxygen atoms into the ion-channel pore, thus conferring a high selectivity for potassium ions over sodium ions [29]. The positive charge at the side chain of the arginine in the G371R variant was predicted to modify the electrostatic potential in the upper part of the channel pore.

**Fig. 2:**
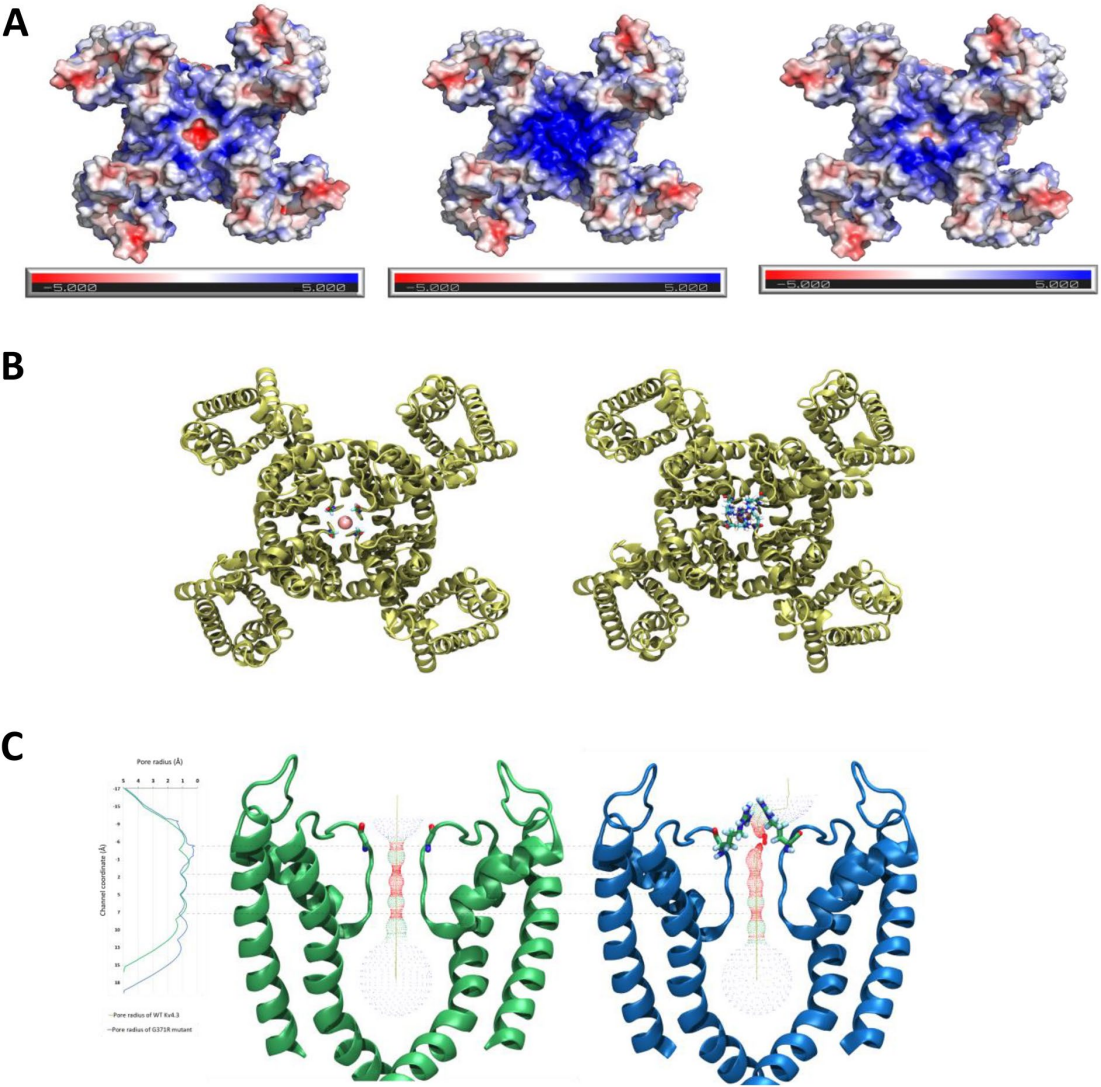
Representation of the electrostatic potential surface and in silico predictions of the changes associated with the G371R variant: (**A**) Electrostatic potential surface of the Kv4.3 (left) channel, the Kv4.3^G371R^ homotetramer (middle), and the Kv4.3 heterotetramer with only one G371R subunit, viewed from the extracellular side. The color scale of the electrostatic potential surface is in units of kT/e at T = 37 °C. Electropositively and electronegatively charged areas are in blue and red, respectively. Neutral residues are white. **B** Pore size comparison for wild-type Kv4.3 (left) vs. Kv4.3^G371R^ homotetramer (right) channels, extracellular view. Intracellular residues from 40 to 180 removed for clarity. **C** The pore radii of the wild-type Kv4.3 (green) and Kv4.3^G371R^ homotetramer (blue) channels were calculated using HOLE. The solvent-accessible pathway along the pore was mapped using the same program. The middle and right images show the wild-type Kv4.3 and Kv4.3^G371R^ homotetramer channels, respectively. The amino acids at the 371 position are displayed as sticks. For clarity, only two diagonally-opposed subunits are shown in each case

Calculation of the electrostatic potential surface for the wild-type channel and the G371R variation was performed using APBS software to solve the Poisson-Boltzmann equations (see Methods). The results show that the electrostatic potential at the outer entry of the selectivity filter changed from a localized negative charge to a positive region (Fig. 2).

Furthermore, due to the longer side chain of arginine compared to glycine, we would expect pore size to be altered, modifying the most probable ion route through the channel. To quantify this change, pore dimensions were analyzed using the HOLE algorithm [17]. The G371R variant's selectivity filter was found to be narrower than that of the wild-type channel, causing the ion conduction pathway to be constricted at its cytoplasmic side. The most constricted point in the variant is located above the S0 coordination site on the selectivity filter, narrowing this region from 2.44 Å to 0.52 Å, and probably collapsing this binding site (see Fig. 2).

Thus, the positively-charged arginine residue introduced at the selective filter in the variation narrowed and electropositively lined the outer part of the pore, hindering potassium ion flux as compared to the wild-type channel without significantly altering the backbone structure.

Next, we aimed to demonstrate a possible dominant effect of the G371R variant in putative heterotetrametric Kv channels. Our reasoning was that since the mutation was present in the proband in a heterozygous state, and functional Kv4.3 channels are formed by the assembly of four subunits, it seemed likely that the G371R variant would co-assemble with wild-type channels, forming heterotetramer channels. We therefore performed electrostatic potential surface calculations of heteromeric channels with one subunit carrying the G371R variant and three wild-type subunits (Fig. 2). The arginine's positive charge neutralized the pore filter's negative charge; consequently, a strong inhibition of potassium ion flux would be predicted.

### Heterologous expression

**Fig. 3:**
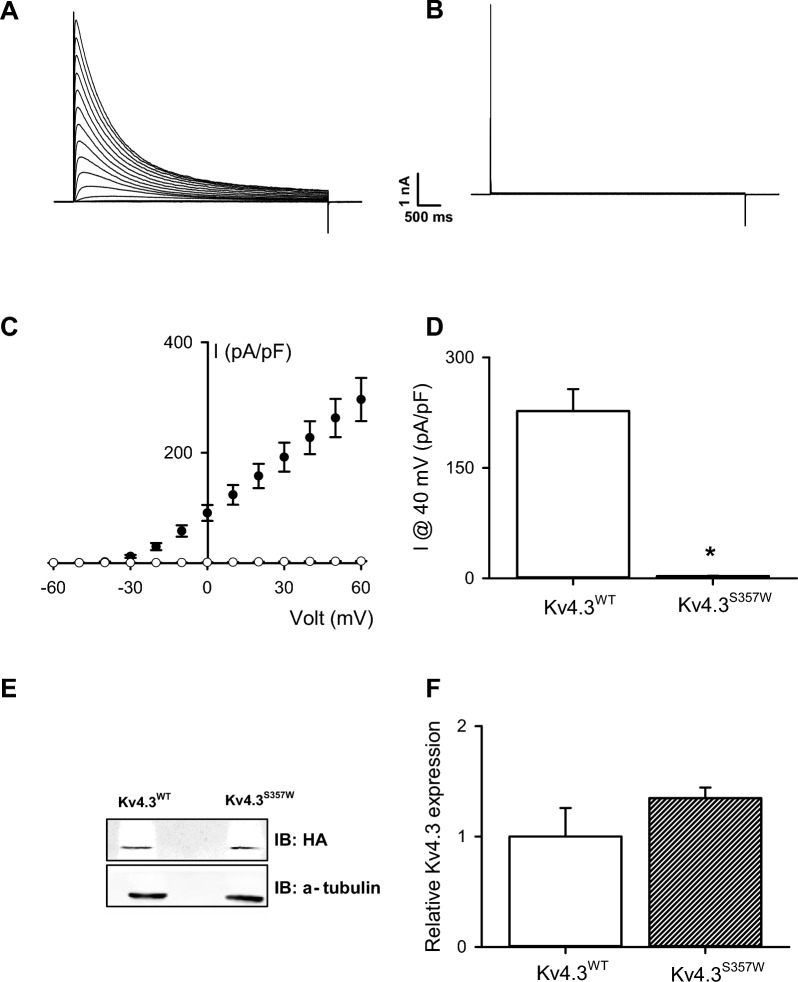
Functional characterization of the S357S variant: Representative K + current traces recorded from cells transfected with the wild-type Kv4.3 (**A**) or Kv4.3^S357S^ channel variant (**B**). Currents were elicited by voltage steps from − 80 to + 80 mV from a holding potential of − 80 mV. Voltage steps lasted 1 s and were applied in 10 mV increments with an interpulse interval of 5 s. **C** Summary peak current I/V plots (mean ± SEM) obtained from currents family as shown in (**A**) and (**B**). The black symbols in the plot represent wild-type Kv4.3 currents while the white symbol stands for the Kv4.3^S357W^ channel variant. The best fit to a Boltzmann equation (see Methods) is represented by a solid line. **D** Summary bar graph showing the normalized peak current amplitudes at + 40 mV (n = 6). **E** Representative immunoblots showing total protein expression of the wild-type Kv4.3 and Kv4.3^S357W^ variant. Cell lysates were subject to immunoblotting analyses with the indicated antibodies. **F** Summary bar graph showing protein density standardized as the ratio of each variant to the corresponding total α-tubulin signal, followed by normalization with respect to the wild-type Kv4.3 as a control (n = 4). *p < 0.01 vs. Kv4.3^WT^

In contrast to the conclusive data from the *in-silico* analysis for the G371R variant, our *in-silico* study for the S357W variant did not provide clear insights into this variant's functional consequences; therefore, we conducted experiments on AD293 cells transfected with either wild-type Kv4.3 (Kv4.3^wt^) or mutant Kv4.3^S357W^ to investigate the effects of this variation on Kv4.3-dependent currents. Potassium currents were recorded by applying voltage pulses from − 80 to 80 mV, and a large transient outward current in Kv4.3^wt^-transfected cells was observed. However, no current was detected in Kv4.3^S357W^-transfected cells (Fig. 3A). To explore whether the observed loss of channel activity was due to enhanced protein degradation, we examined Kv4.3 total protein expression levels and found no change in total Kv4.3^S357W^ protein levels vs. the wild-type counterparts (Fig. 3B). While this result suggests that the mutation does not affect overall protein abundance, it raises the possibility that the mutation may instead alter channel trafficking. Such alterations could result in a lack of potassium current in cells transfected with the mutant version of the channel (Fig. 3).

**Fig. 4:**
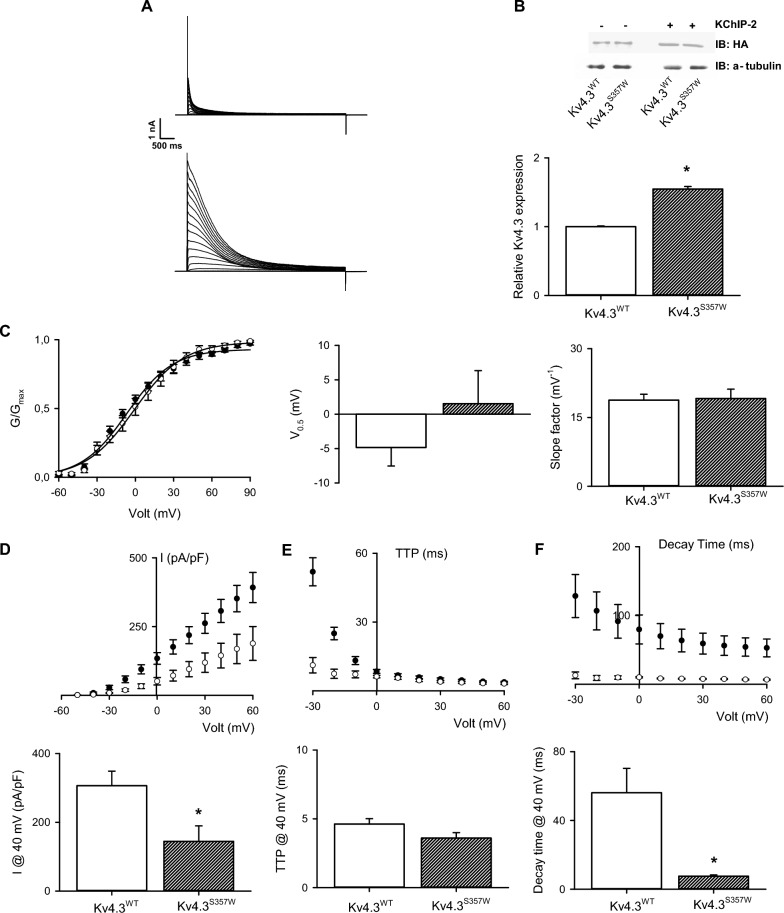
KChIP-2 partially restores the potassium currents of the S357S variant: (**A**) Representative K^+^ current traces recorded from cells transfected with the wild-type Kv4.3 (upper) or Kv4.3^S357S^ (lower) channel and the auxiliary subunit KChIP-2. Currents were elicited by voltage steps from − 80 to + 80 mV from a holding potential of − 80 mV. Voltage steps lasted 1 s and were applied in 10 mV increments with an interpulse interval of 5 s. **B** The upper portion of the figure displays a representative immunoblot showing total protein expression of the wild-type Kv4.3 and Kv4.3^S357W^ variant with and without KChIP-2. Cell lysates were subjected to immunoblotting analyses with the indicated antibodies. The summary bar graph shows protein density standardized as the ratio of each variant to the corresponding total α-tubulin signal, followed by normalization with respect to the wild-type Kv4.3 as a control (n = 4). **C** Left: Normalized G–V plots (mean ± SEM) measured at the peak current from currents family as shown in (**A**). The best fit to a Boltzmann equation (see Methods) is represented by a solid line. Middle: Summary bar graph (n = 6) showing half-activation voltages of normalized conductance, derived from fits of the Boltzmann function to data. Right: Summary bar graph (mean ± SEM) showing the Boltzmann slope factor (n = 6). **D** Summary peak current I/V plots obtained from currents family as shown in (**A**). The black symbols in the plot represent wild-type Kv4.3 currents while the white symbol stands for the Kv4.3^S357W^ channel variant. The best fit to a Boltzmann equation (see Methods) is represented by a solid line. Lower: Summary bar graph showing the normalized peak current amplitudes at + 40 mV (n = 6). **E** Voltage-dependent activation kinetics (mean ± SEM) of wild-type Kv4.3 currents (black symbols) and the Kv4.3^S357W^ channel variant (white symbols) expressed as time to peak (TTP). Lower: Summary bar graph (mean ± SEM) showing the TTP at + 40 mV (n = 6). **F** Deactivation time constant for the inactivating current, derived from the best fit to an exponential decay function. The black symbols in the plot represent wild-type Kv4.3 currents, while the white symbol stands for the Kv4.3^S357W^ channel variant. Lower: Summary bar graph showing the decay time at + 40 mV (n = 6). *p < 0.01 with respect to Kv4.3^WT^ plus KChIP-2

The KChIP2 auxiliary subunit, highly expressed in the brain [30], is known to promote Kv4.3 protein expression and cell surface localization. Therefore, cells were co-transfected with KChIP2 and either the mutant or wild-type Kv4.3 channel. As seen in Fig. 4, co-expression of KChIP2 promoted greater expression of total Kv4.3^S357W^ than Kv4.3^wt^ proteins (Fig. 4B). Nevertheless, although robust outward potassium currents were observed in cells expressing both Kv4.3 channels variants, cells overexpressing Kv4.3^S357W^ displayed a smaller current density compared to Kv4.3^wt^-transfected cells (Fig. 4B). To assess changes in voltage-dependent activation, the normalized conductance was plotted as a function of membrane voltage (Fig. 4C). These data were fitted with a Boltzmann equation to determine changes in the half-activation voltage (V_a_) and activation slope constant (*k*), showing no differences in either constant (Fig. 4D).

To quantitatively compare the voltage-dependent activation and inactivation kinetics of currents from the mutant and wild-type channels, we determined the time to peak (TTP) of the current amplitude and the time taken to reach 63% of peak current as a surrogate for current inactivation. As seen in Fig. 3E, Kv4.3^S357W^ channels reached peak amplitude earlier, indicating a faster activation kinetic at negative membrane potentials. Likewise, mutant channels displayed faster inactivation kinetics throughout the entire voltage range measured (Fig. 4F).

**Fig. 5:**
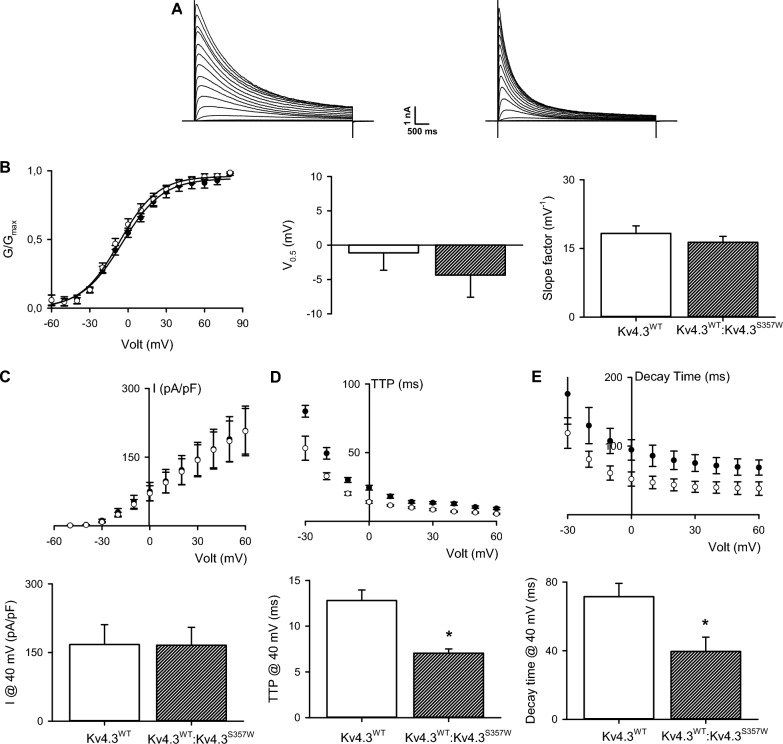
The S357W variant exerts a dominant effect on the functional expression of Kv4.3 channels: (**A**) Representative K^+^ current traces recorded from cells transfected with the wild-type Kv4.3 (left) or wild-type Kv4.3 and Kv4.3^S357W^ channel at 1:1 proportions (right). Currents were elicited by voltage steps from − 80 to + 80 mV from a holding potential of − 80 mV. Voltage steps lasted 1 s and were applied in 10 mV increments with an interpulse interval of 5 s. **B** Left: Normalized G–V plots (mean ± SEM) measured at the peak current from currents family as shown in (**A**). The best fit to a Boltzmann equation (see Methods) is represented by a solid line. Middle: Summary bar graph (n = 6) showing half-activation voltages of normalized conductance, derived from fits of the Boltzmann function to data. Right: Summary bar graph (mean ± SEM) showing the Boltzmann slope factor (n = 6). **C** Summary peak current I/V plots obtained from currents family as shown in (**A**). The black symbols in the plot represent wild-type Kv4.3 currents, while the white symbol stands for the Kv4.3^WT^:Kv4.3^S357W^ channels. The best fit to a Boltzmann equation (see Methods) is represented by a solid line. Lower: Summary bar graph showing the normalized peak current amplitudes at + 40 mV (n = 6). **D** Voltage-dependent activation kinetics (mean ± SEM) of wild-type Kv4.3 currents (black symbols) and Kv4.3^WT^:Kv4.3^S357W^ channels (white symbols) expressed as time to peak (TTP). Lower: Summary bar graph (mean ± SEM) showing the TTP at + 40 mV (n = 6). **E** Deactivation time constant for the inactivating current, derived from the best fit to an exponential decay function. The black symbols in the plot represent wild-type Kv4.3 currents, while the white symbol stands for Kv4.3^WT^:Kv4.3^S357W^ channels. Lower: Summary bar graph showing the decay time at + 40 mV (n = 6). *p < 0.01 with respect to Kv4.3^WT^

Finally, to explore a possible dominant effect of Kv4.3^S357W^ in heterotetrametric Kv channels, we co-expressed Kv4.3wt channels with their mutant counterparts. These experiments were performed in the absence of the KChIP2 auxiliary subunit to prevent Kv4.3^S357W^ homotetramer channels from reaching the plasma membrane and modifying whole-cell current kinetics. As shown in Fig. 5, the mean current amplitude in cells co-transfected with both Kv4.3 channels did not significantly differ from the mean current recorded in cells transfected with the Kv4.3wt channels alone (Fig. 5C). This result suggests that the variant did not have a significant impact on potassium conductance. However, the potassium currents recorded in cells transfected with both types of Kv4.3 channels exhibited a faster decay time and a shorter time to peak when compared to the currents recorded in cells transfected only with the wild-type Kv4.3 channel (Fig. 3). This finding implies that although the variants may assemble, their functional effect on potassium currents is limited to a slight change in current kinetics.

## Discussion

Here, we present four cases of SCA19/22 in Latin American patients, harboring two different *KCND3* missense variants with no previous functional studies. The clinical severity spectrum ranged from a milder late-onset and slowly progressive pure ataxia in the Chilean family carrying the S357W variant to an early-onset ataxia associated with significant neurodevelopmental features in the Mexican patient carrying the G371R variant.

The S357W variant was found in three male relatives, with a segregation pattern consistent with a dominant phenotype. The clinical features of the affected individuals indicated a typical presentation of SCA19, which is known to cause slowly progressive pure ataxia in approximately 65% of adult-onset cases. In this case, the disease had a long-standing duration, with the father surviving over 50 years after disease onset. The case of the Mexican patient was similar to that of a Spanish patient with the same genetic change [26]. In both cases, segregation analysis suggested de novo inheritance and a clinical presentation on the severe end of the SCA19/22 spectrum, with early-onset symptoms of developmental delay (DD), cerebellar ataxia, autism spectrum disorder (ASD), dysarthria, and sialorrhea from infancy [23, 26].

There is an ongoing debate about whether there is a clear genotype–phenotype correlation associated with early and late-onset SCA19/22 manifestations, mainly due to clinical heterogeneity [2, 5]. Our study demonstrates that although each variant impaired Kv4.3 channels when expressed alone (Figs. 2 and 3), the less severe phenotype was associated with a variant that did not impart a functional negative dominant effect on the wild-type channel, as demonstrated by the co-expression experiments (Fig. 5).

Based on the stoichiometry of potassium channels and assuming that each transfected channel shows equal expression, the likelihood of a heterotetrameric channel having at least one mutated subunit is 93%. On the other hand, the probability of wild-type homotetrameric channels is 7%. Therefore, if negative functional dominance occurs, the whole-cell current would presumably fall between these two values when partial dominance is achieved; in other words, if total dominance were achieved, the total current in this co-transfection scenario should be 7%. However, our experimental data indicates no difference in total potassium current whether the wild-type channel is expressed alone or in conjunction with the mutant channel. Nevertheless, the observed changes in current kinetics (Fig. 5) suggest an effective interaction between the two channels when co-expressed. Thus, our data indicates that the S357W interacts with the wild-type subunit to form heterotetramer version of Kv4.3 channels; however, no functional dominance is attained as a consequence of this interaction.

Subtle changes in current kinetics of ion channels have been linked to other neurological disorders [31], and we therefore cannot rule out a functional effect of the S357W variant when assembled with the wild-type subunit. However, the contrast with the effect of the G371R variant, which correlates with a more severe phenotype, is evident. Indeed, the location and type of modification of the G371R variant was shown to dramatically change the surface electrostatic potential at the pore of the Kv4.3 channel, even when only one subunit in the heterotetrametric channel was replaced (Fig. 2). Seminal studies on potassium channels have demonstrated that substituting either glycine in the GYG sequence by a neutral amino acid leads to loss of potassium selectivity [32]. The underlying reason for this phenomenon may be related to the loss of one of the five K^+^ coordination sites formed by the carbonyl oxygens of the TLGYG required for ion selectivity. In fact, ion channels with two or three binding sites become nonselective [33], showing the impact of alternations in this region on potassium channel function, including the glycine substitution in the member of the Mexican family.

Furthermore, the variant mentioned in this study modifies the surface electrostatic potential in the outer region of the pore, as shown in Fig. 2. Studies have demonstrated that charges in the outer vestibule of an ion channel pore can impact conductance by either attracting permeant cations into the vestibule [34] or blocking the channel when the electrostatic potential surface becomes positive due to the protonation of critical residues [35]. Hence, the neutralization of the negative electrostatic potential surface observed here when only one subunit was included in the heterotetrameric channel model (Fig. 2) suggests that, in addition to the expected decrease in selectivity, a reduced conductivity is anticipated with this variant, resulting in a negative functional dominance.

There are approximately 35 variants of the *KCND3* gene that have been linked to human pathology, but only a few of these variants have undergone functional studies [36, 37]. It is noteworthy that gain-of-function mutations, which are associated with cardiac phenotypes such as bradycardia, are mainly located at the channel's C-terminus region [36]. On the other hand, loss-of-function mutations are localized in the S5-S6 linker and pore region of the potassium channel and are associated with cerebellar ataxia [38]. The impact of ataxia-related variants on Kv4.3 potassium channel function ranges from complete to partial loss of function. In addition, not every mutation studied exerts functional dominance over the wild-type channel [37, 38].

The diverse range of functional effects and dominance, which differ among the specific variants, may help explain the wide clinical spectrum observed thus far. However, the experimental data, including the analyses presented here, strongly suggest that the severity of neurological deficits in patients is directly linked to the degree of Kv4.3 channel dysfunction.

Hereditary ataxias are a group of conditions that share their core features (i.e., gait disturbances, visual problems, and dysarthria) but can have different inheritance patterns, associated clinical manifestations, and long-term outcomes. Their cause can be traced to one of many genes. For example, for spinocerebellar ataxias, which have an autosomal dominant inheritance pattern, more than 40 genetic loci have been identified [39]. Repeat expansion mutations cause several of them and have been known for decades.

The advancement of next-generation sequencing has significantly increased access to genetic testing, enabling the detection of single nucleotide variants. However, it is important to use this information carefully in a clinical setting, as interpreting genomic data without proper context can lead to misleading results. Therefore, it is essential to always correlate genetic findings with the clinical context, and whenever feasible, with familial segregation and functional data.

KCND3 was identified as a locus associated with ataxia in 2013. However, the precise geographical distribution of this disease subtype is still not fully understood. The data presented in this study contribute to the global database for future research related to this locus and may serve in future revisions of the diagnostic flowcharts used by clinicians in their decision-making processes. The experimental data and analyses strongly suggest that the severity of neurological deficits in patients is directly linked to the degree of Kv4.3 channel dysfunction, which helps explain the wide clinical spectrum observed to date.

## Language edition and AI use

## Data Availability

Not applicable.
